# Exploring the role of resilience and quality of life in preoperative fear of cancer recurrence among patients with oral and maxillofacial cancer: A cross-sectional study

**DOI:** 10.1371/journal.pone.0339329

**Published:** 2026-01-06

**Authors:** Jingya Yu, Yu Zhang, Zhixiang Chen, Xuemei Yang, Lu Bai, Lixia Kuang, Fenglian Li, Xiaoqin Bi

**Affiliations:** 1 State Key Laboratory of Oral Diseases & National Center for Stomatology & National Clinical Research Center for Oral Diseases and Dep. of Orthognathic and TMJ Surgery, West China Hospital of Stomatology, Sichuan University, Chengdu, China; 2 West China School of Nursing, Sichuan University, Chengdu, China; 3 Division of Head & Neck Tumor Multimodality Treatment, Cancer Center, West China Hospital, Sichuan University, Chengdu, China; 4 School of Nursing, Chongqing Medical University, Chongqing, China; 5 Department of Stomatology, Longhua People’s Hospital of Shenzhen, Guangdong, Shenzhen, China; 6 Department of Hepatobiliary, Chongqing Fuling Hospital, School of Medicine, Chongqing University, Chongqing, China; 7 Department of Head and Neck Oncology, West China Hospital of Stomatology, Sichuan University, Chengdu, China; King Saud University / Zagazig University, EGYPT

## Abstract

**Aims:**

Fear of cancer recurrence (FCR) is a common concern among cancer survivors; however, its prevalence and determinants in patients with oral and maxillofacial cancers have not been thoroughly investigated. This study aimed to assess the severity of FCR and explore the relationships among resilience, quality of life (QoL), and FCR in preoperative oral and maxillofacial cancer patients.

**Methods:**

A cross-sectional survey was conducted in tertiary hospitals in China from March to July 2024. The study collected demographic and clinical data, the Fear of Cancer Recurrence Inventory-Short Form (FCRI-SF), the 10-item Connor-Davidson Resilience Scale (CD-RISC-10), and the University of Washington Quality of Life Questionnaire (UW-QOL). Data analysis included descriptive statistics, independent samples t-tests, one-way ANOVA, and multiple linear regression analysis.

**Results:**

The study included 281 preoperative oral and maxillofacial cancer patients. The mean FCRI-SF score was 17.38 (SD = 4.73), with 85.1% of the participants exhibiting high FCR. Resilience, as measured by CD-RISC-10, had a mean score of 23.33 (SD = 3.85), whereas QoL, measured by the UW-QOL, had an average of 58.36 (SD = 8.61). Correlation analysis revealed significant associations among age, sex, first treatment, resilience, QoL, and FCR. Multiple linear regression revealed that QoL and sex were significant predictors of FCR, explaining 40.1% of the variance (*P* < 0.001). Higher QoL was associated with lower FCR, and males reported lower levels of FCR than females did.

**Discussion:**

Preoperative oral and maxillofacial cancer patients experience elevated levels of FCR, which are influenced by various socio-demographic and clinical factors. These findings highlight the need for healthcare providers to develop targeted interventions and comprehensive support systems to address FCR effectively in this high-risk group.

## 1 Introduction

Oral and maxillofacial cancers are the sixth most prevalent malignancies globally [1,2]. Annually, more than 600,000 individuals are diagnosed with head and neck cancers, with oral cancer accounting for approximately 400,000 of these cases, underscoring a marked and concerning increase in incidence. Oral and maxillofacial cancers encompass a spectrum of malignancies affecting anatomical sites such as the lips, tongue, floor of the mouth, buccal mucosa, gums, hard and soft palate, maxilla (upper jaw), mandible (lower jaw), and the oropharynx [3]. Despite advances in surgery, radiotherapy, and chemotherapy, the 5-year survival rate remains unsatisfactory (47.1%–64.6%), with recurrence rates ranging from 10% to 50% [4–6]. Given this substantial recurrence risk, patients frequently experience profound psychosocial distress, including depression, suicidal ideation, and fear of cancer recurrence (FCR) [7]. However, few studies have specifically explored FCR during the preoperative phase, when uncertainty about treatment and prognosis is greatest.

Fear of cancer recurrence (FCR), a negative psychological experience that persists during and after treatment, is defined as fear, anxiety, and worry about the potential recurrence or progression of cancer, either in the same organ or elsewhere in the body [8]. It is conceptually distinct from general surgical anxiety: the latter concerns the impending operation (e.g., anesthesia, postoperative pain), whereas FCR reflects an enduring, anticipatory fear of recurrence, even before treatment begins [9]. This distinction is clinically relevant, as FCR may persist throughout the disease trajectory, influencing medical decision-making and adherence to treatment [10]. Excessive FCR can drive patients to pursue unnecessary procedures or frequent follow-ups, whereas fatalistic beliefs may lead others to delay or refuse treatment, ultimately compromising clinical outcomes.

Epidemiological evidence highlights that FCR is among the most prevalent and distressing psychological concerns in oncology, with 22%-87% of cancer survivors reporting moderate-to-high levels [11,12]. Extensive evidence from breast and colorectal cancer cohorts indicates that FCR often endures well beyond curative treatment, disrupting emotional regulation, compromising treatment adherence, and diminishing long-term quality of life [13]. Similarly, recent research among HNC survivors has shown that FCR remains highly prevalent, with elevated levels particularly observed in patients experiencing functional impairments, visible disfigurement, or greater emotional distress related to appearance and communication difficulties [14]. However, preoperative patients, especially those undergoing their initial treatment, are particularly susceptible as they confront the uncertainty surrounding the future course of their cancer. The fear of recurrence is often exacerbated by their lack of familiarity with the treatment process and the uncertain nature of potential outcomes [15]. Elevated FCR has been associated with greater psychological distress, poorer quality of life (QoL), and increased healthcare utilization [16–18]. Yet, empirical evidence on preoperative FCR in oral and maxillofacial cancers remains limited.

Resilience, defined as the ability to adapt positively to adversity and maintain psychological well-being, has gained increasing attention in oncology as a key factor in coping with the stressors associated with cancer diagnosis and treatment [19,20]. Patients with oral and maxillofacial cancers often confront disfigurement, speech impairment, and loss of social confidence, making resilience crucial for maintaining emotional stability and adaptive functioning [21]. Evidence suggests that variations in resilience among cancer patients can lead to significant differences in their quality of life and ability to cope with the disease [22,23]. Moreover, research has shown that individuals exhibiting resilience tend to achieve more effective psychological adaptation in response to both acute and chronic stressors [24]. However, most studies have focused on posttreatment survivors, leaving a gap in understanding resilience and QoL in shaping preoperative FCR.

Guided by stress-coping theory, this study conceptualizes resilience as a protective factor and QoL as an indicator of perceived functional and emotional well-being. We hypothesize that higher resilience and better QoL are associated with lower preoperative FCR. This study aims to fill a critical gap by examining the interplay among resilience, QoL, and FCR in preoperative oral and maxillofacial cancer patients. Findings are expected to inform targeted psychosocial interventions and guide early, multidisciplinary strategies to enhance psychological preparedness before surgery.

## 2. Method

### 2.1 Study design and participants

This cross-sectional study was conducted between March and July 2024, and involved participants from the Department of Head and Neck Oncology, West China Hospital of Stomatology, Sichuan University. Informed consent was obtained from all participants prior to their inclusion in the study. An overview of the study design is presented in the graphical abstract (S1 Fig).

Eligible participants met the following criteria: (1) were 18 years of age or older, (2) had a confirmed diagnosis of oral and maxillofacial cancers in the preoperative phase, (3) were aware of their disease diagnosis and condition, (4) were able to complete the questionnaire independently or with assistance from caregivers, and (5) provided voluntary consent to participate in the study. Participants were excluded if they met any of the following conditions: (1) had other serious chronic illnesses such as heart, kidney, or lung failure; such advanced comorbities are known to substantially diminish QoL and elevate psychological distress, thereby introducing confounding effects on the relationships between FCR, resilience, and QoL that this study sought to investigate [25,26]; (2) had a history of mental illness or disturbances of consciousness; or (3) had incomplete data.

### 2.2 Sample size and sampling strategy

Guidelines for multiple-factor analysis recommend a sample size of at least 5–10 times the number of variables [27]. In this study, with 17 variables, including 11 demographic characteristics, 1 variable related to fear of cancer recurrence, 3 resilience variables, and 2 quality of life variables the minimum required sample size was calculated to be between 85 and 170 participants. To account for an anticipated 20% attrition rate, we adjusted the target sample size to between 102 and 204 participants. Ultimately, 330 oral and maxillofacial cancer patients were recruited through a convenience sampling method from this hospitals, and 281 patients with complete data were available for analysis.

### 2.3 Data collection

Ward nurses employed the established inclusion and exclusion criteria to identify eligible participants through the convenience sampling method. A registered nurse and a postgraduate nursing student administered the surveys. Before commencing the survey, nurses provided participants or their caregivers with clear instructions on the use of a QR code to access and complete the electronic questionnaire. Data were collected via the widely recognized online survey platform Questionnaire Star, developed by Changsha Ranxing Information Technology in China. Each survey was conducted at the patient’s bedside and typically took 5–10 minutes to complete.

Because all questionnaire items were self-administered by patients and investigators were responsible solely for providing standardized instructions and checking for completeness, formal inter-rater reliability metrics (e.g., Cohen’s Kappa or the intraclass correlation coefficient) were not applicable to this study design. Instead, methodological rigor was ensured through a one-week structured training program for all investigators covering eligibility screening, participant instruction, and data-recording procedures. During data collection, investigators adhered to a unified standard operating protocol under the oversight of a senior nurse, and periodic random audits were conducted to verify data accuracy and completeness.

#### 2.3.1 Demographic characteristics.

The demographic characteristics included age, sex, residential location, monthly income (in RMB, yuan), marital status, educational level, primary caregiver, and type of medical insurance. The clinical characteristics include the site of the disease, duration of illness, and presence of cancer-related pain.

#### 2.3.2 Fear of cancer recurrence.

Fear of cancer recurrence was assessed using the 9-item Fear of Cancer Recurrence Inventory-Short Form(FCRI-SF), a concise version of the full 42-item FCRI [12]. This study utilized the validated Chinese version of the FCRI-SF [28]. The participants rated their fear of cancer recurrence on a 5-point Likert scale, ranging from 0 (never) to 4 (always), with total scores ranging from 0-36. Higher scores reflect greater levels of fear. An FCRI-SF total score above 13 has been identified as a clinically significant cutoff for fear of cancer recurrence, suggesting the need for additional psychological support or intervention [29]. The Chinese version of the FCRI-SF has excellent internal consistency, with a Cronbach’s alpha coefficient of 0.912 [30].

#### 2.3.3 Resilience.

To evaluate resilience, this study utilized the 10-item Connor-Davidson Resilience Scale (CD-RISC-10). The scale consists of three factors: Tenacity, Strength, and Optimism [31]. The CD-RISC-10 is a condensed version of the original 25-item CD-RISC, selected for its brevity and efficiency [32]. Each item is rated on a 5-point scale, ranging from 0 (not true at all) to 4 (true nearly all the time), with higher total scores indicating greater resilience; lower CD-RISC-10 scores represent weaker resilience and may help guide psychosocial strategies aimed at enhancing coping skills and stress management in vulnerable patients [33]. The Chinese version of the CD-RISC-10 was employed to evaluate resilience within the Chinese context [34]. The scale demonstrated strong internal consistency, with a Cronbach’s alpha of 0.91, confirming its reliability for measuring resilience in the Chinese population [34].

#### 2.3.4 Quality of life.

Quality of life was assessed using the University of Washington Quality of Life Questionnaire (UW-QOL), designed for individuals with oral and maxillofacial cancer. This questionnaire evaluates the symptoms experienced by participants over the previous seven days [35]. The UW-QOL comprises two primary domains: physical function, which includes aspects such as chewing, swallowing, speech, taste, saliva, and appearance, and social-emotional function, which encompasses factors such as anxiety, mood, pain, activity, recreation, and shoulder function [36]. The scale consists of 12 disease-specific items, each with 3–5 response options. Each option is assigned a score ranging from 0 to 100, with higher scores reflecting a better quality of life [37].

### 2.4 Data analysis

Data analysis was conducted using the Statistical Package for the Social Sciences (SPSS), version 26.0 (IBM Corporation, Armonk, NY, USA). Descriptive statistics, including frequencies, percentages, means, and standard deviations (SD), were computed. To identify relevant determinants of FCR, one-way analysis of variance, independent t-tests, and Pearson’s correlation analysis were employed.

Variables significant in univariate analyses (p < 0.05), along with clinically relevant covariates (tumor stage and treatment history), were entered into a multiple linear regression model to adjust for potential confounding effects.

Given the limited sample size within several clinical subgroups (e.g., tumor stage, tumor site, treatment history), stratified analyses were not performed because subgroup regression would have lacked adequate statistical power and produced unstable estimates. Instead, clinically relevant variables (tumor stage and treatment history) were adjusted as covariates in the multivariable model to account for their potential effects on FCR.

### 2.5 Ethical considerations

This study was approved by the Ethics Committee of West China Hospital of Stomatology, Sichuan University (Grant No. WCHSIRB-CT-2024–107). All procedures were conducted in accordance with the principles outlined in the Declaration of Helsinki. Written informed consent was obtained from all participants prior to data collection.

## 3. Results

### 3.1 Demographic characteristics

**Fig 1:**
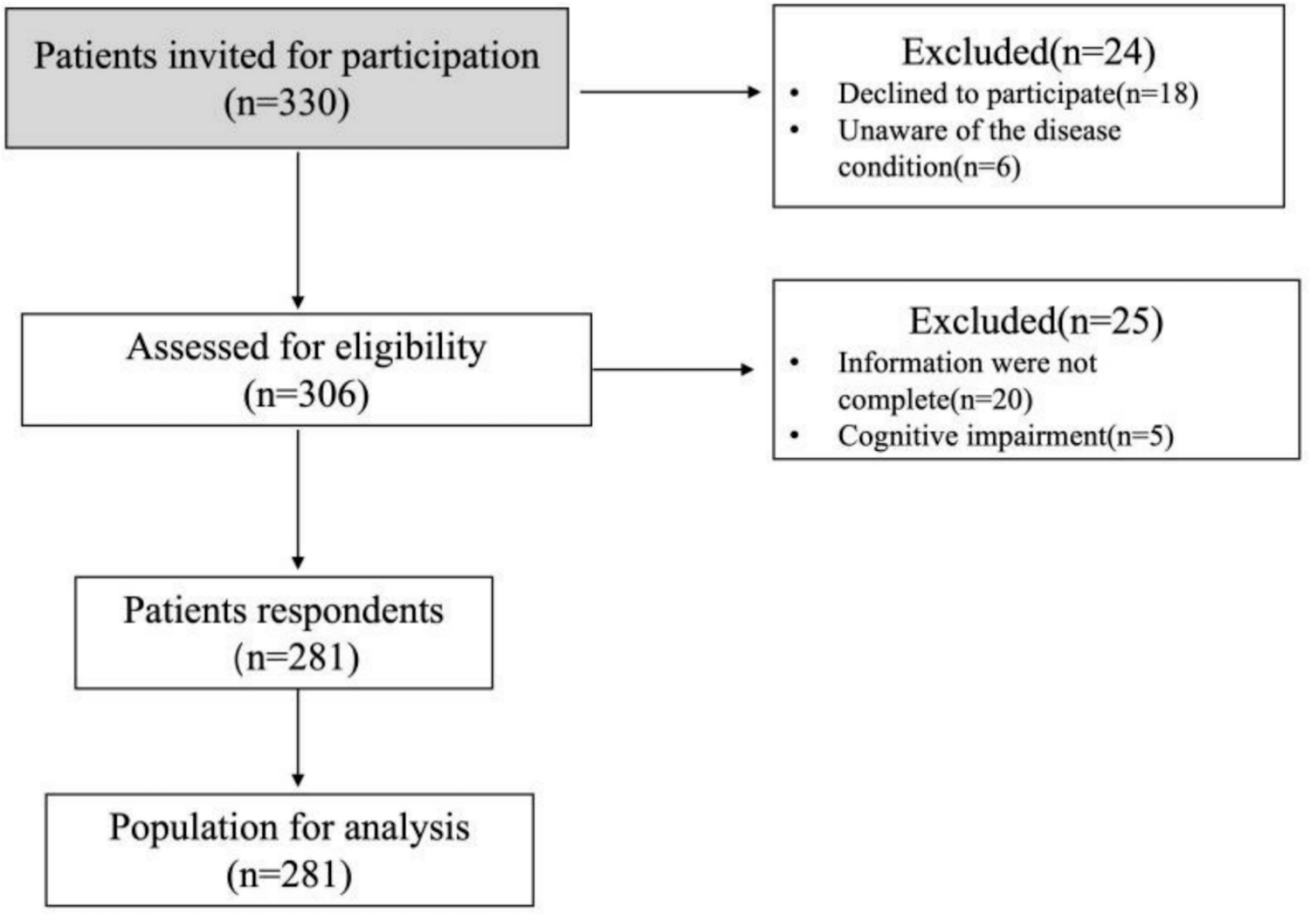
Flowchart of participant recruitment.

A total of 281 patients were screened to meet eligibility criteria (Fig 1). The demographic and clinical characteristics of the participants are summarized in Table 1. The participants ranged in age from 18 to over 60 years, with 38.8% being 60 years or older. The majority of participants were female (50.9%) and lived in urban areas (41.3%). Educational attainment was predominantly at the primary school level (70.8%), and a significant portion of the sample reported a monthly income of less than ¥2000 (70.1%). Most participants were married (92.9%), and nearly half were retired (49.5%). In terms of clinical staging, 14.2% of patients were Stage I, 23.1% Stage II, 30.2% Stage III, and 32.4% Stage IV. The vast majority of patients (approximately 90%) were diagnosed with squamous cell carcinoma, whereas a small proportion had other histological subtypes, such as mucoepidermoid carcinoma or adenoid cystic carcinoma.

**Table 1:** General information of the participants and analysis of FCRI-SF scores (n = 281).

Independent variable	*N*	*%*	Mean (*SD*)	*t/F*/r	*P*
**Age**					
18-60	172	61.2	18.36(5.01)	4.795	**0.000**
≥60	109	38.8	15.83(3.79)		
**Sex**					
Female	143	50.9	19.47(4.64)	8.449	**0.000**
Male	138	39.1	15.22(3.76)		
**Location**					
Village	113	40.2	17.31(4.84)	0.238	0.788
Town	52	18.5	17.79(4.67)		
City	116	41.3	17.27(4.68)		
**Income**					
<¥2000	197	70.1	17.16(4.45)	0.778	0.507
¥2000-5000	64	22.8	18.17(5.10)		
¥5000-10000	15	5.3	17.00(6.50)		
≥¥10000	5	1.8	17.00(4.84)		
**Education**					
Primary school	199	70.8	17.12(4.45)	1.324	0.267
Secondary school	42	14.9	18.52(5.40)		
College	37	13.2	17.68(5.38)		
Diploma or above	3	1.1	15.00(2.64)		
**Employment**					
Employed	95	33.8	17.41(4.86)	1.423	0.243
Unemployed	47	16.7	16.36(4.84)		
Retired	139	49.5	17.71(4.59)		
**First treatment**					
Yes	155	55.2	16.59(4.67)	−3.17	**0.002**
No	126	44.8	18.36(4.64)		
**Site of cancer**					
Tongue	85	17.40(4.46)	57.82(8.40)	0.317	0.866
Cheek	45	17.42(5.19)	57.59(8.24)		
Mandible	44	18.00(5.00)	57.15(8.15)		
Sublingual	21	16.76(4.02)	55.26(6.06)		
Others	86	17.17(4.83)	59.06(8.17)		
**Pain**					
Yes	159	56.6	17.65(4.74)	1.106	0.270
No	122	43.4	17.02(4.71)		
**Stage**					
Stage I	40	14.2%	15.80 (4.10)	1.24	0.297
Stage II	65	23.1%	16.90 (4.45)		
Stage III	85	30.2%	17.55 (4.68)		
Stage IV	91	32.4%	18.40 (4.90)		
**UW-QOL**			58.36(8.61)	−0.518	**0.000**
**CD-RISC-10**			23.33(3.85)	−0.220	**0.000**

Abbreviations: **FCRI-SF**,Fear of Cancer Recurrence Inventory-Short Form; **CD-RISC-10**,10-item Connor-Davidson Resilience Scale; ***SD***, standard deviation.

Of the 330 patients invited, 24 declined or were unaware of their diagnosis, and 25 were excluded due to incomplete data or cognitive impairment. A total of 281 participants were included in the final analysis.

### 3.2 Analysis of the differences in FCRI-SF scores

The analysis of the FCRI-SF scores across various demographic and clinical variables revealed several significant correlations (Table 1). Age and sex were significantly associated with FCR scores, with participants aged 18−60 exhibiting markedly higher FCR scores than those aged ≥60 years (*P* < 0.001). Similarly, compared with male participants, female participants presented significantly elevated FCR scores (*P* < 0.001). The participants who underwent their first treatment reported significantly lower FCR scores than did those with previous treatment experience (*P* < 0.001). Moreover, both quality of life (UW-QOL) and resilience (CD-RISC-10) were inversely correlated with FCR (*P* < 0.001).

Socioeconomic variables, including income, education, and employment status, did not show statistically significant associations with FCR in univariate analyses (all p > 0.05), and therefore were not retained for multivariable modeling.

### 3.3 Psychological, resilience, fear of cancer recurrence, and quality of life scores

Table 2 presents the scores for psychological resilience, fear of cancer recurrence, and quality of life among the study participants (n = 281). The FCRI-SF revealed a mean score of 17.38 (SD = 4.73), with scores ranging from 4 to 31. Notably, 85.1% of the participants scored above the clinically significant cutoff of 13, indicating a high prevalence of FCR in this population. The CD-RISC-10 had a mean score of 23.33 (SD = 3.85) with scores ranging from 15 to 39 points, this score reflects a relatively low-to-moderate level of resilience among the participants. The mean UW-QOL score was 58.36 (SD = 8.61), with scores ranging from 38 to 79. The scores for the physical function domain ranged from 33 to 79, with a mean of 56.51 (SD = 8.03), reflecting a moderate level of physical function impairment. In contrast, the social-emotional function domain had a mean score of 60.20 (SD = 12.12), with scores ranging from 33 to 88, suggesting that while some participants experienced significant emotional and social challenges, others reported relatively better emotional well-being.

**Table 2:** FCRI-SF, CD-RISC-10, and UW-QOL scores (n = 281).

Variable	Range	Mean	*SD*
**FCRI-SF, points**	4-31	17.38	4.73
**CD-RISC-10, points**	15-39	23.33	3.85
**UW-QOL, points**	38-79	58.36	8.61
Physical function	33-79	56.51	8.03
Social-emotional function	33-88	60.20	12.12

Abbreviations: FCRI-SF, Fear of Cancer Recurrence Inventory-Short Form; CD-RISC-10, 10-item Connor-Davidson Resilience Scale; UW-QOL, University of Washington Quality of Life Questionnaire; SD, standard deviation.

### 3.4 Comparison of scores across FCRI-SF Items

The violin plot effectively visualizes the distribution of scores across the nine FCRI-SF items, illustrating the unique score distributions for each item (S2 Fig). Items such as FCR1, FCR4, and FCR6 exhibit broader distributions, indicative of greater variability, whereas FCR3 demonstrates a narrower spread, suggesting a more concentrated range of scores around the mean. The mean score for FCR5 is notably higher than that for the other items, highlighting its tendency to receive higher overall scores. The white box within each violin represents the interquartile range (IQR), capturing the middle 50% of the data, with the black horizontal line and dot marking the median score. The shape and width of each violin reflect the density of scores, where wider sections correspond to higher concentrations of responses.

### 3.5 Factors influencing FCR

Following the univariate analysis, variables that showed statistical significance were included in a multiple linear regression analysis to further explore their impact on FCR (Table 3). The regression model identified two significant predictors of FCR. The model explained a substantial portion of the variance in FCR scores, with an R² of 0.401 and an adjusted R² of 0.388 (F = 30.634, *P* < 0.001). Quality of life (UW-QOL) was found to be negatively associated with FCR (β = −0.391, *P* < 0.001,95% CI for B: −0.271 to −0.158), indicating that patients with a higher quality of life experience less fear of cancer recurrence. Sex also emerged as a significant factor influencing FCR (β = −0.313, *P* < 0.001, 95% CI for B: −3.895 to −2.026). Specifically, the negative coefficient suggests that male patients tend to have lower FCR scores than female patients do, highlighting the role of sex differences in the level of fear of cancer recurrence. The standardized beta coefficients (β) and R² values represent the effect sizes, indicating that both predictors had moderate-to-strong effects on FCR and jointly explained approximately 40% of its variance.

**Table 3:** Factors associated with FCRI-SF scores among patients (n = 281).

Variable	*B*	SE-B	*β*	*t*	*P*	95% *CI*
**Constant**	33.628	1.821		18.467	0.000	30.043 - 37.213
**UW-QOL**	−0.215	0.029	−0.391	−7.491	0.000	−0.271 - −0.158
**Sex**	−2.961	0.475	−0.313	−6.237	0.000	−3.895 - −2.026

Abbreviations: *B*, unstandardized coefficient; *β*, standardized beta coefficient; *CI*, confidence interval; SE-B, standard error of ***B. R²* = 0.401; adjusted *R²* = 0.388; *F* = 30.634; *P* < 0.001.**

## 4. Discussion

This study is the first to examine the prevalence and associated factors of FCR among preoperative oral and maxillofacial cancer patients, demonstrating that this population experiences elevated levels of FCR. FCR was highly prevalent in this population, with QoL and sex identified as significant predictors, underscoring the complex and multifactorial nature of patients’ psychological responses.

Although the majority of existing research on FCR has concentrated on post-treatment or survivorship populations, evidence pertaining to the preoperative phase remains scarce. This phase constitutes a distinct period of psychological vulnerability, during which patients experience anticipatory anxiety regarding treatment outcomes, functional impairment, and potential disfigurement, concerns that differ fundamentally from the experiential fears encountered after treatment [38]. Notably, existing FCR research in breast, colorectal, and head and neck cancer has predominantly focused on postoperative survivors who have already experienced treatment-related functional changes and recurrence monitoring [14,18]. In contrast, preoperative patients face anticipatory rather than experiential fears, underscoring a distinct and previously underexamined psychological stage. Investigating FCR prior to surgery provides critical insight into early psychological responses and underscores the importance of proactive screening and timely psychosocial intervention.

The relatively high prevalence of FCR and low-to-moderate resilience may be partly attributable to socioeconomic vulnerabilities. Limited education, low income, and caregiving burdens among older or retired patients can restrict access to psychosocial resources, intensify anxiety about disease progression, and hinder adaptive coping. In today’s era of widespread internet use, the abundance of cancer-related information may further exacerbate uncertainty and intensify fears of recurrence. Previous oncology research demonstrates that socioeconomic disadvantage is linked to greater psychological distress, lower resilience, and elevated FCR, particularly among patients confronting complex treatment demands [39]. Individuals with lower income or limited education may have diminished access to medical care and fewer coping resources, thereby amplifying fears of recurrence. Recognizing these factors is crucial for healthcare providers to deliver balanced education and develop targeted interventions that alleviate psychological distress and support informed decision-making. It is noteworthy that in our sample, socioeconomic indicators such as income and education were not statistically associated with FCR in univariate testing. This suggests that their impact may not manifest directly in the preoperative phase, or that additional contextual or mediating factors, beyond the scope of the present dataset, may shape the relationship between socioeconomic status and FCR. Future studies incorporating more granular socioeconomic measures may provide further insight into these dynamics.

This study revealed that preoperative oral and maxillofacial cancer patients presented an average FCR score of 17.38 (SD = 4.73), notably above the standard mean. This elevated score likely reflects the patients’ heightened anxiety about potential treatment-related issues, including facial tissue defects, impaired swallowing and chewing functions, altered dietary habits, and alterations in speech. Research focusing on preoperative FCR emphasizes that such concerns are central to patients’ emotional experiences and can influence their psychological well-being before any treatment takes place [40]. Consistent with prior international research, which reported that 30%-35% of head and neck cancer patients experience dysfunctional FCR [41]. Item 5 (“I believe that I am cured and that cancer will not come back”) received the highest score, suggesting ambivalence between optimism and fear. Within the context of Chinese cultural avoidance of death-related discussions, culturally sensitive education that normalizes these concerns and emphasizes treatment progress is essential. Meanwhile, this education should emphasize advancements in treatment and the potential for positive outcomes. Additionally, facilitating culturally sensitive support groups can help mitigate isolation, foster shared experiences, and strengthen resilience within the community. Moreover, a high level of social support is widely acknowledged as a critical external resource for cancer patients, helping to sustain a positive emotional state and functioning as a psychological buffer against stress [42]. Such support empowers cancer patients to face the disease with resilience. Therefore, healthcare professionals and family caregivers need to provide comprehensive social support throughout the treatment and follow-up stages.

In the univariate analysis, resilience was negatively correlated with FCR, suggesting that higher resilience was associated with lower FCR. However, after adjustment for QoL and sex in the multivariate model, resilience was no longer a statistically significant predictor. This indicates that resilience may not independently predict FCR once patients’ quality of life is taken into account, and its apparent effect in unadjusted analyses should be interpreted with caution. While the influence of resilience may be intertwined with patients’ physical and emotional functioning, the present data do not allow conclusions regarding mediation or causal pathways. Clinically, this finding underscores the need for integrated interventions that strengthen resilience while simultaneously addressing QoL and functional recovery. Programs incorporating structured psychosocial counseling, goal-oriented rehabilitation, and family or peer support are likely to achieve greater psychological and functional benefits than resilience training alone. Such multidimensional approaches can enhance adaptive coping, restore daily functioning, and ultimately mitigate the intensity and persistence of FCR among patients with oral and maxillofacial cancers.

Our findings revealed a strong negative association between QoL and FCR, suggesting that poorer QoL is closely linked to heightened recurrence-related fears. Among these domains, physical functioning and emotional well-being emerged as the most influential contributors to FCR. Physical impairments, such as dysphagia, speech difficulties, and facial disfigurement, can serve as persistent reminders of the illness, thereby intensifying recurrence-related fears. In parallel, emotional distress and reduced social connectedness may weaken patients’ ability to tolerate uncertainty and mobilize coping resources. These domain-specific patterns highlight that the interplay between physical dysfunction and emotional vulnerability plays a central role in shaping preoperative FCR. From a clinical perspective, these findings underscore the need for multidisciplinary interventions that integrate medical, rehabilitative, and psychosocial care. Practical interventions such as targeted counseling, cognitive-behavioral therapy (CBT), and mindfulness can be tailored to the unique challenges faced by oral and maxillofacial cancer patients [43,44]. For example, CBT techniques may be adapted to address appearance-related distress and communication anxiety by incorporating structured modules that target concerns about facial asymmetry, postoperative scars, unintelligible speech, drooling, or oral malodor, all of which are common sources of social avoidance in oral and maxillofacial cancer patients. In addition, preoperative functional rehabilitation strategies, such as Shaker exercises and Mendelsohn maneuvers, may help preserve swallowing function, enhance oropharyngeal muscular coordination, and strengthen patients’ sense of control before surgery [45]. Moreover, to enhance early identification of psychological risk, routine FCR screening should be integrated into the preoperative workflow. Administering a brief FCRI-SF assessment during surgical counseling may help to identify high-risk individuals, such as younger patients, women, and those with poorer quality of life, as indicated by our findings. In addition, incorporating caregiver-reported perceptions of recurrence-related worries may reveal unexpressed or suppressed fears, providing a valuable complementary perspective for comprehensive preoperative assessment.

Our study also revealed significantly higher levels of FCR among female patients, a finding consistent with previous research in other cancer populations [46,47]. Women’s greater vulnerability to recurrence-related fears may derive from their multiple familial and professional roles, heightened emotional sensitivity, and stronger self-image awareness. In oral and maxillofacial cancers, where treatment often alters facial appearance and functional capacity, these factors can exacerbate concerns about social stigma and body image. Clinically, this underscores the importance of sex-sensitive psychological interventions. Incorporating Acceptance and Commitment Therapy (ACT) may foster self-acceptance, enhance psychological flexibility, and alleviate appearance-related distress [48]. Complementary body-image–focused rehabilitation and gender-informed psychoeducation can further promote emotional regulation, self-efficacy, and sustained engagement in recovery.

In the correlation analysis of this study, younger patients (under 60 years of age) were found to have significantly greater levels of FCR, which also reported an association between sex and FCR, though it focused on patients after treatment [49]. This elevated FCR in middle-aged and younger individuals may be linked to their substantial family and societal responsibilities, including the dual demands of raising children and caring for elderly relatives. Moreover, the psychological impact of a cancer diagnosis on younger patients can be profound, leading to difficulties in accepting their condition and potentially intensifying their fear. Conversely, older patients, while experiencing a reduction in social roles, benefit from greater life experience. In the context of China’s rich traditional culture, Confucian philosophy, which emphasizes the principles of “facing” and “accepting” [50], may facilitate older patients’ adaptation to the disease and its related changes. To mitigate the elevated FCR observed in middle-aged and younger patients, it is recommended that nurses implement targeted psychological interventions, including individualized therapy, stress management techniques, and coping strategies. Additionally, involving family members and caregivers through educational programs can reduce patient stress by assisting in managing familial responsibilities. Furthermore, organizing workshops that offer practical guidance on balancing responsibilities during treatment, alongside strategies for effective time management and self-care, can provide critical support in helping patients navigate their challenges.

This study examined FCR and its associated psychosocial factors among preoperative oral and maxillofacial cancer patients. However, several limitations must be acknowledged. First, the cross-sectional design precludes causal inference and prevents characterization of how FCR evolves across the perioperative trajectory. Clinically, this limits our ability to determine when psychological distress peaks and when interventions, such as resilience training or FCR-focused counseling, should be optimally delivered. Future longitudinal studies tracking patients from diagnosis through postoperative recovery are needed to clarify FCR trajectories and to guide the timing and tailoring of stage-specific psychosocial interventions. Second, the use of convenience sampling from a single country (China) may restrict cultural and clinical generalizability. Larger, cross-regional cohorts would help validate whether the observed predictors of FCR apply across diverse healthcare systems. Third, certain clinically relevant variables, such as detailed clinicopathological indicators and standardized assessments of preoperative functional status, were not uniformly available and thus could not be included in the analysis, potentially constraining the interpretability of the regression model. Finally, although the FCRI-SF is a validated instrument, it may not fully capture the multidimensional nature of FCR. Future studies using comprehensive FCR measures and qualitative approaches could provide deeper insight into patients’ experiences and support the development of more precisely targeted interventions.

### Conclusion

In summary, this study is the first to examine fear of cancer recurrence and its psychosocial correlates among preoperative oral and maxillofacial cancer patients, offering novel insights into how resilience and quality of life interact to influence FCR before treatment. The findings underscore the need for multidimensional, phase-specific interventions, including resilience enhancement, functional rehabilitation, and sex-sensitive counseling, to mitigate recurrence-related fears and promote holistic recovery. From a clinical perspective, these results emphasize the value of early psychological screening during the preoperative stage, when anticipatory fears and functional concerns are most pronounced. Future longitudinal studies are warranted to delineate the trajectory of FCR across the treatment continuum and to determine the optimal timing and intensity of interventions (e.g., preoperative vs. postoperative) that can sustain long-term psychological well-being.

## Supporting information

S1 Fig Graphical abstract. This graphical abstract provides an overview of fear of cancer recurrence (FCR) among 281 patients with oral and maxillofacial cancers in China, showing assessment tools, prevalence of high FCR, and significant predictors.
(DOCX)

S2 Fig Distribution of scores across FCRI-SF items. This figure illustrates the distribution patterns of scores across the nine items of the Fear of Cancer Recurrence Inventory–Short Form (FCRI-SF), each representing a specific dimension of patients’ concerns regarding cancer recurrence.
(DOCX)
