# Supplementary material for: Exploring the role of resilience and quality of life in preoperative fear of cancer recurrence among patients with oral and maxillofacial cancer: A cross-sectional study
Source: PLoS One. 2026 Jan 6;21(1):e0339329. doi: 10.1371/journal.pone.0339329 (PMC12773800; doi:10.1371/journal.pone.0339329)
Supplement: S1 Fig — This graphical abstract provides an overview of fear of cancer recurrence (FCR) among 281 patients with oral and maxillofacial cancers in China, showing assessment tools, prevalence of high FCR, and significant predictors. (DOCX) [file pone.0339329.s001.docx]

**
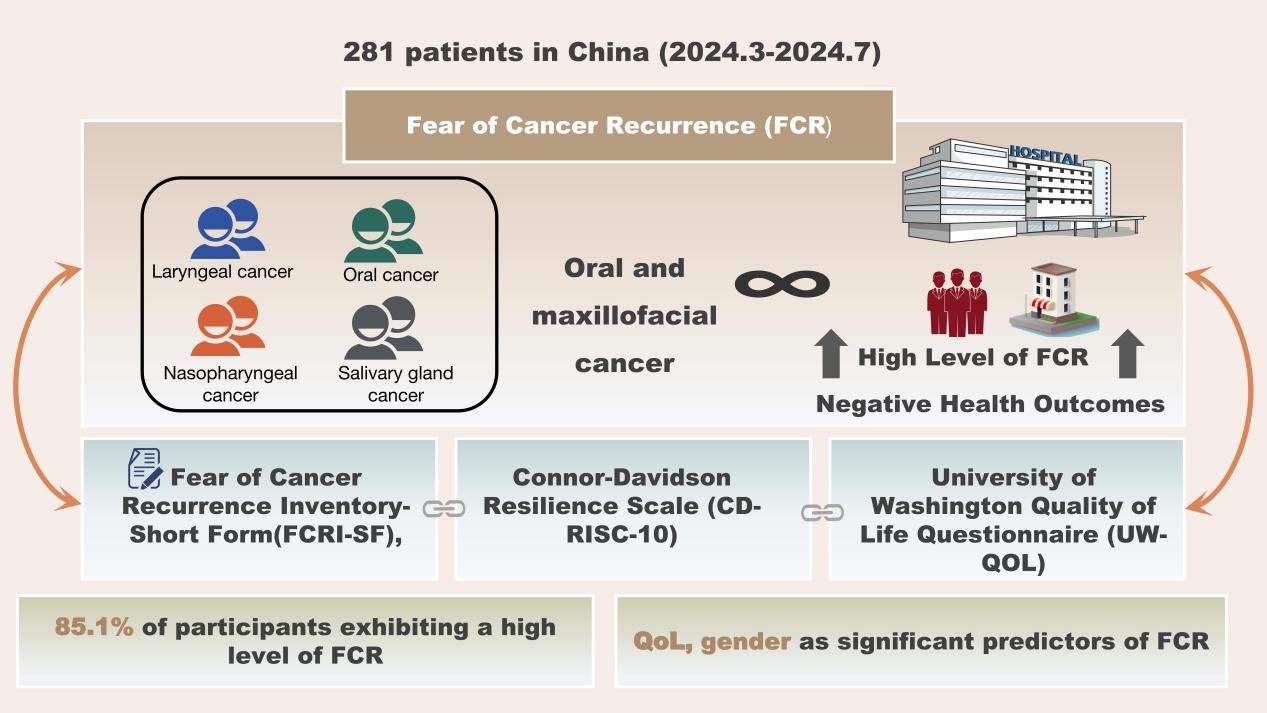
**

**Figure S1. Graphical Abstract**

**Figure caption:** Overview of fear of cancer recurrence (FCR) among 281 patients with oral and maxillofacial cancers in China. The study assessed FCR using the FCRI-SF, resilience using the CD-RISC-10, and quality of life using the UW-QOL. A high level of FCR was observed in 85.1% of participants, with QoL and gender identified as significant predictors of FCR.
