# Supplementary material for: Exploring the role of resilience and quality of life in preoperative fear of cancer recurrence among patients with oral and maxillofacial cancer: A cross-sectional study
Source: PLoS One. 2026 Jan 6;21(1):e0339329. doi: 10.1371/journal.pone.0339329 (PMC12773800; doi:10.1371/journal.pone.0339329)
Supplement: S2 Fig — This figure illustrates the distribution patterns of scores across the nine items of the Fear of Cancer Recurrence Inventory–Short Form (FCRI-SF), each representing a specific dimension of patients’ concerns regarding cancer recurrence. (DOCX) [file pone.0339329.s002.docx]

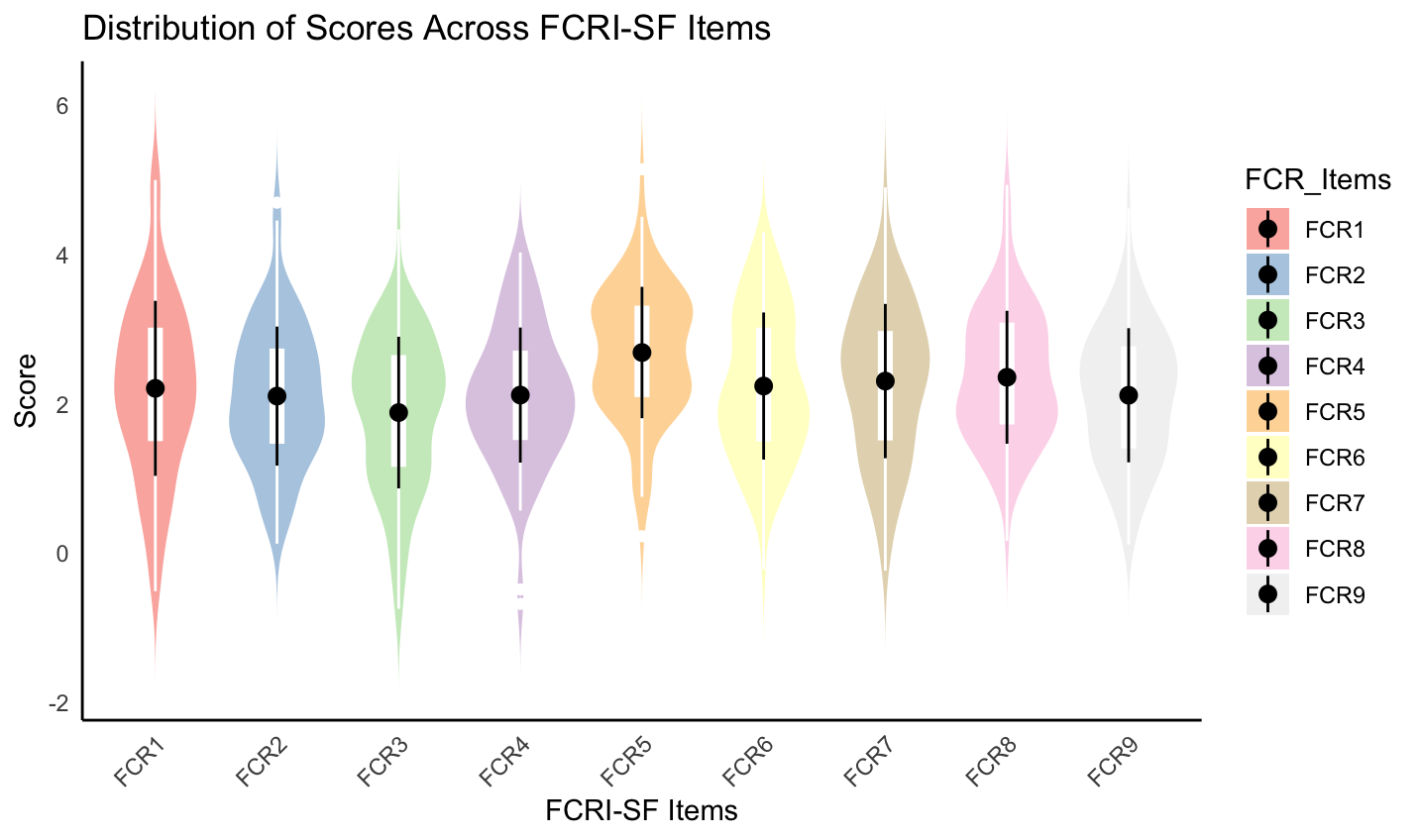


**Figure S2. Distribution of Scores across FCRI-SF Items**

**Figure Caption:** The figure presents the nine items of the FCRI-SF, which assess various dimensions of fear of cancer recurrence. Each item is designed to evaluate specific aspects of patients' concerns and anxieties regarding cancer recurrence：

**Item1:** I am anxious or worried about the possibility of cancer recurrence; **Item 2:** I am afraid of cancer recurrence;

**Item 3**: I believe that being worried or anxious about cancer recurrence is normal;

**Item 4**: When you think about cancer recurrence, other unpleasant thoughts or images come to my mind (e.g., death, suffering, family consequences);

**Item 5:** I believe that I am cured and that cancer will not come back; **Item 6:** Do you believe that you are at risk of cancer recurrence?

**Item 7:** Do you often think about the possibility of cancer recurrence? **Item 8:** How much time per day do you spend thinking about the possibility of cancer recurrence?

**Item 9:** How long have you been thinking about the possibility of cancer recurrence?
